# Supplementary material for: A drop in Sahara dust fluxes records the northern limits of the African Humid Period
Source: Nat Commun. 2019 Aug 23;10:3803. doi: 10.1038/s41467-019-11701-z (PMC6707271; doi:10.1038/s41467-019-11701-z)
Supplement: Supplementary file 1 — Supplementary Information [file 41467_2019_11701_MOESM1_ESM.pdf]

Supplementary Notes for:

A drop in Sahara dust fluxes records the northern limits of the African Humid Period

Daniel Palchan[1,2,*] and Adi Torfstein[1,2]

[1] The Fredy & Nadine Herrmann Institute of Earth Sciences, The Hebrew University of Jerusalem, The Edmond J. Safra Campus, Givat Ram, Jerusalem 91904, Israel. [2] Interuniversity Institute for Marine Sciences, Eilat 88103, Israel. * Corresponding author: Daniel.palchan@mail.huji.ac.il

Supplementary Note 1: Airflow patterns and the rain- dust correlation.

To constrain the particle sources we used a lagrangian model "LAGRANTO" by ETH Zurich, http://iacweb.ethz.ch/staff//sprenger/lagranto/[1], to compute the air flow that carries the dust. For each core site, we calculated the 72 hours back trajectory for a full year every 72 hours. The results for arbitrarily chosen years (1982, 1997, 2005 and 2016) display similar patterns between each other are presented in Figure. S1. The main dust transport routes are summarized in Figure 3 as yellow arrows. It should be noted that the calculated back trajectories of dust sources in the Red Sea region are corroborated by the Nd and Sr isotopic compositions of the sediments and potential source areas of dust[2].

In addition, as terrigenous material from the Horn of Africa carries both mineral dust and terrigenous leaf wax thought to trace the hydrological cycle, their comparison can be used to evaluate the connection between dust and precipitation. We compare the leaf wax values with dust accumulation rates in both cores KL15 and KL11. The central Red Sea is used for comparison because precipitation in the Horn of Africa and the adjacent regions, which supply dust to the central Red Sea[2], show similar trends[3] and are affected by the Western African Monsoon system[4]. Because of inconsistencies in the timing of available samples between data sets, we created an interpolated record of the leaf wax δD and compared it with the dust accumulation rates in the Gulf of Aden and in the central Red Sea.
The results show a good correlation for the core KL11 R^2=0.6 and weaker correlation to core KL15 R^2=0.3 (Fig. S3), confirming that higher precipitation is associated with lower dust accumulation rates. This most likely reflects increased vegetation cover that inhibits dust uplift in the source region, in combination with wet deposition of the dust before it reaches the core site.

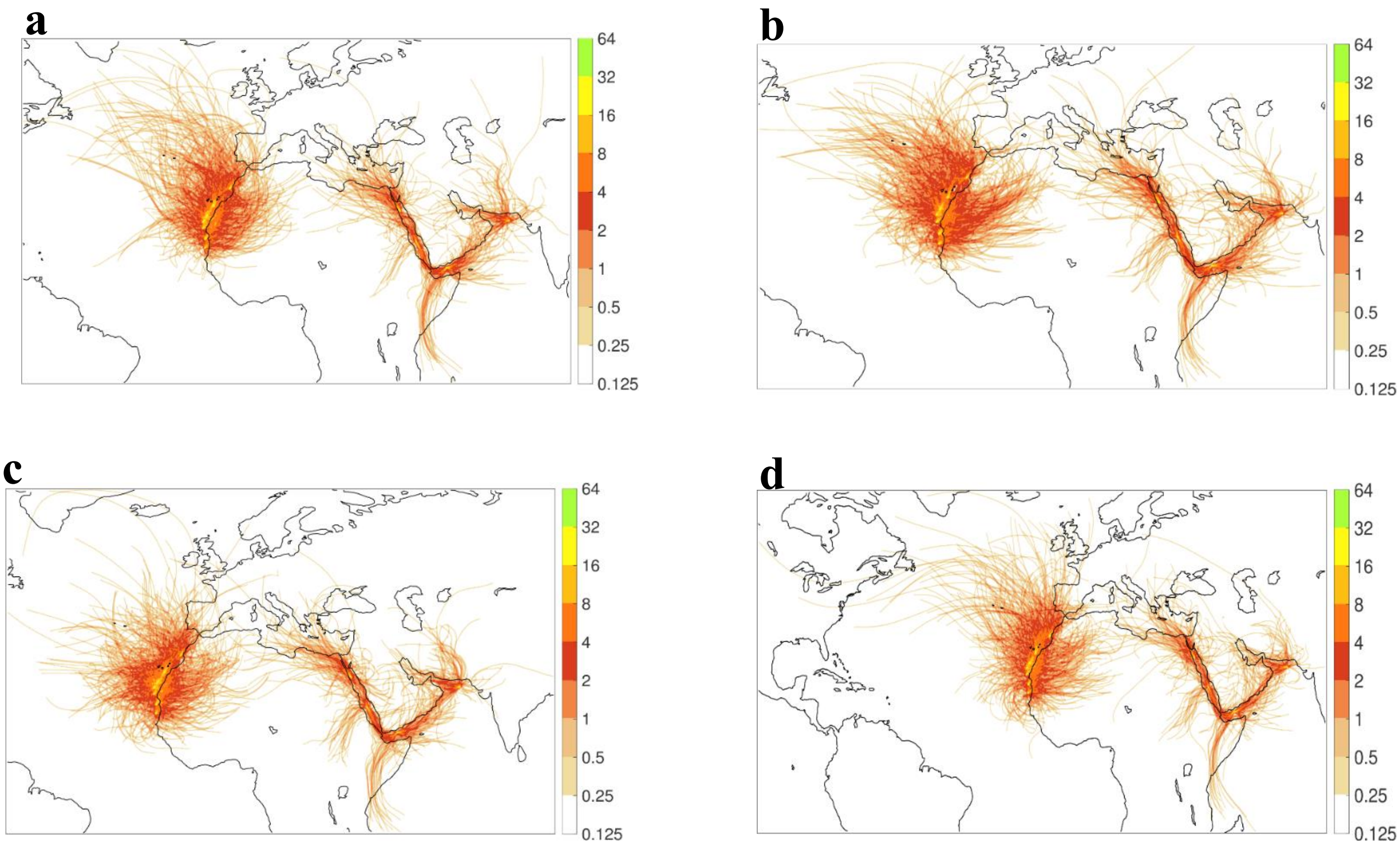

Supplementary Figure 1| One year back the trajectories from the different core sites. Four, arbitrarily chosen years are presented: **a**; 1982, **b**; 1997, **c**; 2005 and **d**; 2016. The trajectories computed every 72 hours using LAGRANTO (http://iacweb.ethz.ch/staff//sprenger/lagranto/) 72 hours backwards. The potential dust transport routes are summarized in Figure 3.

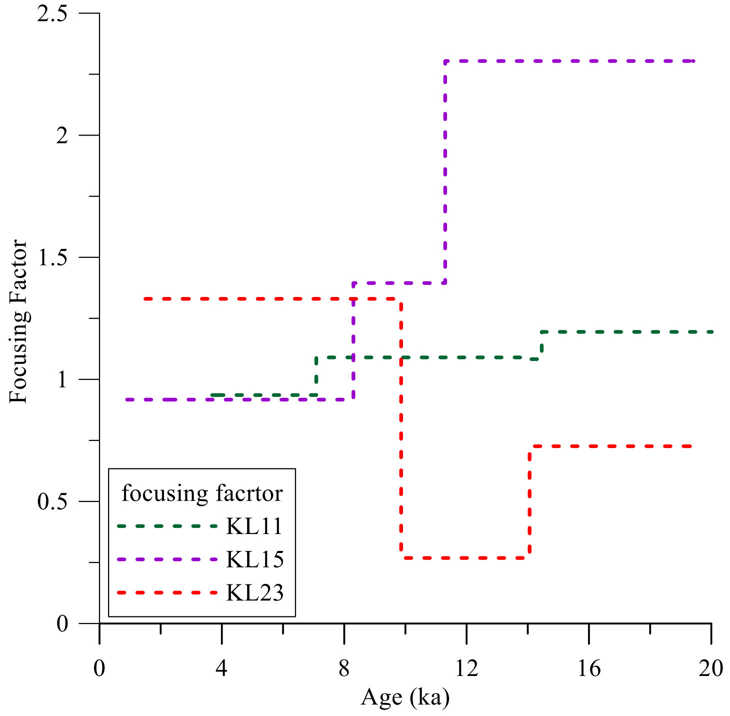

Supplementary Figure 2| Focusing factors of the three cores studied here. Values suggest minimal sediment redistribution in the core sites.

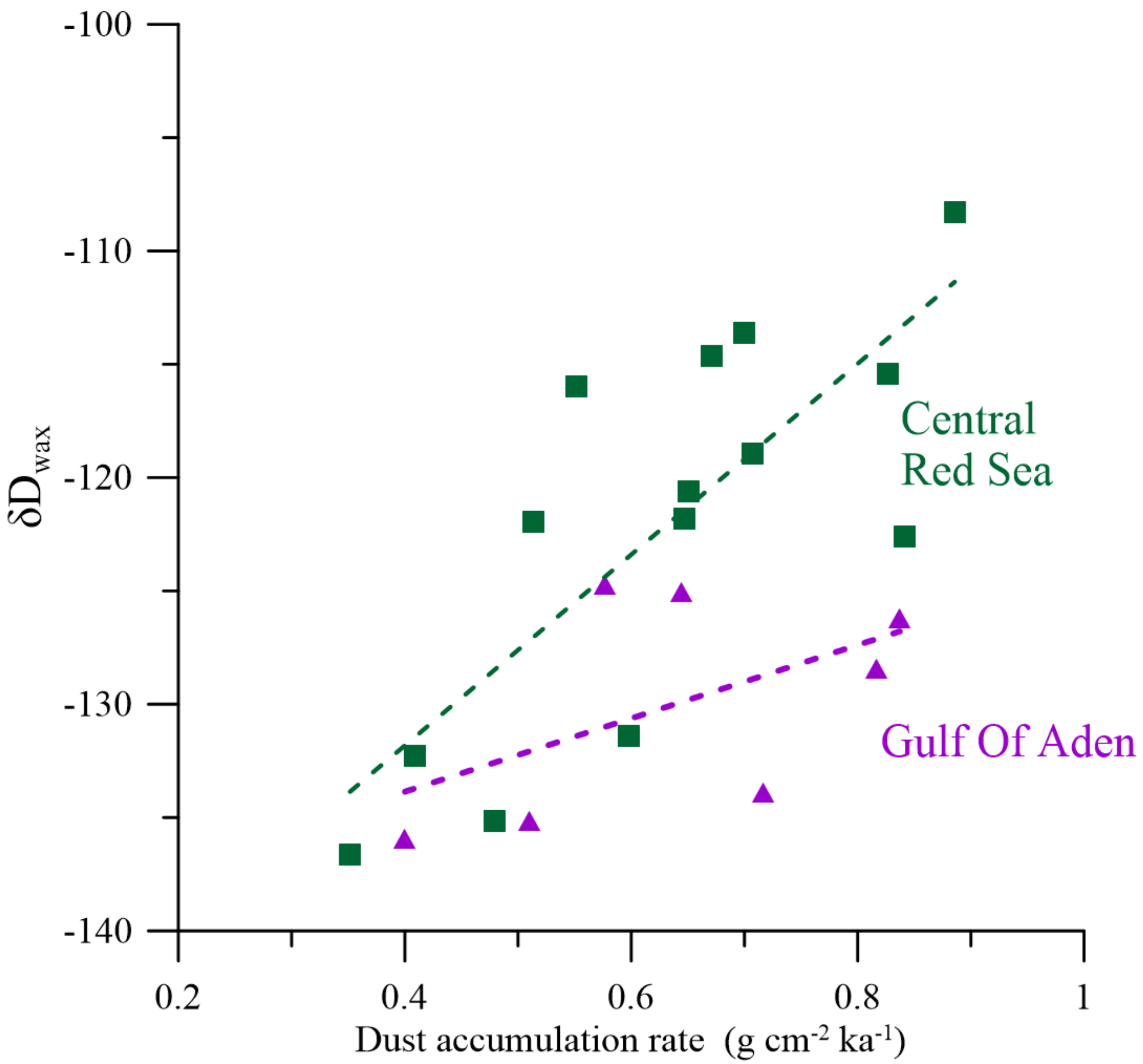

Supplementary Figure 3| Rain and dust correlations in eastern Africa. Comparison between δD_{wax} values in core RC09[4] that reflects precipitation in the Horn of Africa for the past 20 ka, and coeval dust accumulation rates reported here in the Gulf of Aden (KL15) and the central Red Sea (KL11).

Supplementary Note 2: End member analysis for KL23

The terrigenous material deposited at KL23 comprises a mixture of dust particles that have eroded from granitoids of the Saharan shield and the Arabian Nubian Shield[2]. The different crystallization ages of each of these principal sources results with significantly different $^{143}Nd/^{144}Nd$ values that can be used to differentiate between the sources. Both of these sources (i.e., the erosion products) are blown to site KL23 in the Red Sea from northern Saharan deserts, in Libya and Egypt (Fig. S1). In modern dust samples collected in Israel, the dust storm tracks were traced by satellite imagery and atmospheric models, and then compared to the geochemical signal[5]. Palchan et al.[5] identified that dust originating from the Libyan coast with a maritime course before reaching Israel (i.e., not passing over land collecting additional dust) had a geochemical signal of the erosion products of the Saharan shield. However, dust storms originating at Libya that passed over the western desert of Egypt (i.e., deflating dust from different origins), displayed a geochemical signal of a mixture between the two types of granitoids.

We implement a simple model to evaluate the contribution of each granitoid source to the dust deposited at the northern Red Sea using the Nd isotopes that are expressed as:

$$\varepsilon\text{Nd} = \left[\frac{\frac{^{143}\text{Nd}}{^{144}\text{Nd}}_{\text{meas.}}}{\frac{^{143}\text{Nd}}{^{144}\text{Nd}}_{\text{CHUR}}} - 1 \right] x\ 10{,}000$$

whereby CHUR = 0.512638 [6].

Assuming εNd end member compositions of –2 and –12 for Arabian Nubian Shield (ANS) late Proterozoic granitoids and Sahara mid Proterozoic granitoids, respectively, we calculated the relative content of Sahara dust in each sample based on the following mixing equation:

$$\%\ Sahara = \frac{\varepsilon\text{Nd}_{\text{sample}} - \varepsilon\text{Nd}_{\text{ANS}}}{\varepsilon\text{Nd}_{\text{sahara}} - \varepsilon\text{Nd}_{\text{ANS}}}\ x\ 100$$

The results are reported in Supplementary Table 1 and depicted in Figure 2d. During the AHP, the contribution of Sahara dust component to KL23 shows a minor decrease from 38% to 33% (Supplementary Table 1). This small drop in values could be the result of some flow at the Kufrah River[7] that originated from the Tibesti mountains (~20°N) and carried the volcanic rocks comprising the Tibesti complex. Volcanics from Tibesti could drive the εNd to higher values[8], which would drive the Saharan type component to lower values in our end member analysis.

Hence, during the AHP precipitation in the Sahara Desert must have extended up to 20°N, the southernmost reach of the Kufrah watershed, in order to facilitate the small increase we identify in the εNd values.

Supplementary Note 3: Groundwater recharge of northern sites

The AHP has been thoroughly investigated in various sites across the Sahara Desert by modeling and in-situ sampling[8–47]. Based on dust fluxes from the Atlantic Ocean and the Red Sea, which were reconstructed from sediment cores, we draw the line of direct precipitation during the AHP north of the 50mm/y increased precipitation reconstructed by PMIP3 models[48]. Yet, some of the northern most sites - Farfara and Dakhla oases in eastern Africa and Mellala and Hasi el Mejnah in western Africa - record palustrine environments during the early Holocene. Evidence from these sites however, is based on physical structures reflecting groundwater outflow rather than direct precitation[20,24,31,32]. Indeed, these sites are located in areas where groundwater is shallower relative to the surroundings[49]. Furthermore, the watershed of these sites extends significantly southward, and hence, the in situ hydrological changes could reflect environmental changes that occurred hundreds of km away from the sites (Fig. 3):

(1) The western sites watershed reaches the M'zab. Mts. (~150 km to the west), at elevations >1000m and could very well be influenced by Mediterranean source as suggested by recent modeling reconstructions[50,51].

(2) The eastern sites watershed (i.e., Farafra and Dakhla oases) extends south to ~20°N. Thus, evidence for increased hydrological activity in these sites during the AHP likely reflects, at least partially, increased groundwater activity driven by southern precipitation.

Supplementary Note 4: Environmental records and proxies discussed in this study.

We hereby provide a brief overview of the background relating to the records and proxies discussed in this work, which were used for the reconstruction of northern Africa environmental conditions during the AHP. While some of the details are redundant with other descriptions in this manuscript, they are presented here for the purpose of providing the reader with a clear list of the main approaches discussed in this work. The reader is referred to the listed references for a more detailed discussion of each of the records or proxies.

Dust fluxes: Dust fluxes reflect environmental conditions over large areas by averaging wind frequency, soil moisture and overall aridity. Dust accumulation rates in western Africa show significant drop during the AHP[52,53]. This points to more humid conditions that inhibited dust uptake from the central Sahara westward at the same interval. The observations show that this interval was more humid between 20°N and 28°N as core GC27 (Fig. 1) shows minimal decrease in dust fluxes, furthermore the timing is not tuned to the AHP. Dust fluxes in the Arabian Sea suggest wetter conditions prevailed in Arabian dust sources during the AHP as well[54]. Similarly, dust accumulation rates in Eastern Africa, presented in this work, suggest that during the AHP wetter conditions prevailed in eastern Africa between 12°N and 22°N.

Pollen records: Pollen assemblages in terrestrial sites reflect the environmental conditions of past plant growth (available moisture and seasonal temperatures). Several sites from northeast Africa depict that during the early to mid-Holocene wetter conditions prevailed in the currently arid desert[55]. Mainly based on occurrences of Tamarix charcoals, the border between steppe and dessert vegetation was marked at about 23°N[56].

Oxygen isotopes in marine microfossils: The oxygen isotope composition of the G. ruber alba in sediment core extracted from the Nile Delta fan reflects the sea water salinities. Negative values point to increased deliveries of fresh water from the River Nile. During the AHP the oxygen isotope values of this

species in the Nile Delta became significantly lighter, pointing to enhanced outflow from the River Nile, which watershed extends to the Sahel and clearly suggests wetter conditions in Eastern Africa during the AHP[8].

% Saharan Shield dust in KL23: The terrigenous fraction in core KL23 in the northern Red Sea consists two siliciclastic source end members: granites from the Arabian Nubian Shield and granites from the Saharan Shield[2]. The former can be transported to KL23 site by either eolian or fluvial activity whereas the latter is only delivered through eolian processes. Using the measured εNd compositions of the sediments we calculated the relative contribution from each source and found that during the AHP there is a negligible decrease in the eolian Saharan source (<5%). This suggests that the source regions of the dust, in northern Sahara, remained arid throughout the AHP.

Terrigenous grain size in KL23: Fining of terrigenous grain sizes in the Red Sea sediments was shown to be related to fluvial contribution, which in turn points to more humid conditions in the vicinity of the studied site[2,57]. In KL23 at the northern Red Sea no such grain size fining is recorded for sediments of the early Holocene, thus, the conditions surrounding the northern Red Sea remained arid during the AHP.

Terrigenous grain size in KL11: The addition of fine material to the sediments in KL11, central Red Sea, during the AHP suggests wetter conditions prevailed in the neighboring watersheds that supplied it. We suggest the material was derived from the Baraka basin as it is the largest watershed in the vicinity and core KL11 is located under its mouth. Moreover, the addition of fluvial material to KL11 terrigenous sediments dictates that the reported dust accumulation rates are not only of eolian origin. In turn, this suggests that the eolian dust fluxes eastward from Africa decreased by more than 50% as calculated from the data, thus, the 50% dust decrease during the AHP is a minimum estimation.

KL09 Hematite concentration: Hematite content in sediments of the Red Sea and the Mediterranean is linked to concentration of eolian dust[58,59]. At core KL09, central Red Sea, there is a drop in the hematite content that gradually increases into the Holocene. This drop is linked to decrease in the dust deposition and in turn dust deflation off of East Africa.

Gulf of Aden leaf wax δD: Isotope composition of deuterium in leaf wax recovered from sediment core RC09 is a measure of the wetness in the source region, the Horn of Africa[4]. The leaf wax δD record suggests that the Horn of Africa was wetter than present during the AHP. The wetness inhibited dust deflation, as expressed by dust accumulation rates that is observed in core KL15 (Fig. 2).

Grotte de Piste speleothems: This is a karstic cave in northwest Africa with speleothem growth[60]. The $\delta^{18}O$ composition of speleothems that developed in this karstic cave in northwest Africa does not support distinct wet conditions above the cave and its vicinity for the duration of the AHP. Furthermore, COSMOS model simulations[61], suggest that any precipitation that took place, is linked to the north Atlantic oscillation and mid latitude cyclones rather than to the modulation of insolation variations and monsoonal precipitation.

Nd isotopes in core 293G: The isotopic composition of Nd in the terrigenous fraction of the sediments in the Alboran Sea shows variations in the sediment sources[62]. If the AHP monsoonal rains reached northern Sahara, it would be excepted that the Saharan dust component decreased in sync with insolation changes and more intense monsoon activity. Given that this is not the case, it is concluded that dust deflation regions in northwest Africa remained mostly arid though the AHP.

Atlantic sediment cores leaf wax δD: These records show that Western Africa experienced wetter conditions during the AHP[50]. However, we argue that the northern record of core GC27 is biased due to

the mountainous regions of the Atlas Mts. There, the high terrain and proximity to the Atlantic sourced moisture by mid latitude cyclone activity causes the leaf wax isotopic composition of deuterium to reach uniquely low values.

Hassi el Mejnah and Sebkha Mellala: These two water bodies are located at the eastern flanks of the M'zab Mts. Lithological variations from eolian sands to laminated carbonates with gastropods indicate variation in their water balance. Studies performed on the diatom and gastropod assemblages and their stable isotope geochemistry suggest that during the AHP these sites held more water. Whether the source of these wasters is direct precipitation or groundwater recharge was answered using distribution of trace elements in the calcites deposited from them. It was then argued that the source is favorably ground water recharge[10]. The watershed of these sites that are recharged from the Great Western Erg aquifer is at the M'zab Mts.[40], that are related to North Atlantic systems rather than the African monsoon (i.e., similar to cave Grotte de Piste).

Mega Lakes Chott, Fezzan, Ahmet, Darfur, and Chad: These five presumed mega lakes, scattered over northern Africa, were recently re-evaluated in the context of their paleohydrologic history. Based on comprehensive geomorphic observations, Quade et al.[48]concluded that only the southern Lake Chad could be considered to be a mega lake. The others were found to be localized manifestations of groundwater swamps and springs.

Farfara and Dakhla oases: Oases sites at the north eastern flanks of the Sahara Desert. Remnant signs of water presence have been identified at these sites. However, these do not indicate a standing waterbody but rather, sloping wetlands and localized springs[20]. Importantly, the genesis of these waterbodies does not require direct precipitation, and they could be induced by groundwater outflow[31], with distant recharge areas located closer to the equator[63]. In addition, most of the travertines and related sediments identified from these sites were recently dated to the last glacial, preceding the AHP[63].

Wadi Sannur: A speleothem cave located at northeast Africa with sample ages determined to be from previous interglacials MIS 9, 7 and 5e, interpreted to represent increased direct precipitation above the cave during previous interglacials[64]. Importantly, none of the samples found here were from the Holcoene, suggesting that relatively dry conditions prevailed at this site during the AHP.

Selima oases: Buried lake sediments occupying a local depression that were dated to the early Holocene. These include high organic content in mud deposits and relatively low $CaCO_3$ deposition. Furthermore, pollen and diatom assemblages support the occurrence of wetter conditions in the vicinity during the AHP. Hence, it is likely that relatively increased direct precipitation occurred in the region of Selima oases during the AHP[44].

Supplementary Table 1- Siliciclastic grainsizes and Nd isotopes of dust in Red Sea cores.

Core	Depth in core (cm)	Age (ka)	grain size mode (μm)	$^{143}Nd/^{144}Nd$	εNd	% Sahara shield dust
KL23	5	1.5	27	0.512184	-8.8	81
KL23	10	2.7	20	0.512297	-6.7	60
KL23	15	3.0	31	0.512274	-7.1	64
KL23	20	4.8	36	0.512307	-6.5	58
KL23	25	7.0	36	0.512346	-5.7	50
KL23	30	9.9	40	0.512324	-6.1	54
KL23	35	12.0	36	0.512307	-6.5	58
KL23	40	13.3	32	0.512310	-6.4	57
KL23	45	13.4	24	0.512279	-7.0	63
KL23	50	13.5	20	0.512283	-6.9	62
KL23	55	13.6	36	0.512268	-7.2	65
KL23	60	14.0	33	0.512261	-7.4	67
KL23	65	14.1	13	0.512309	-6.4	57
KL23	70	16.0	22	0.512300	-6.6	59
KL23	75	16.1	26	0.512316	-6.3	56
KL23	80	16.6	25	0.512314	-6.3	56
KL23	80	16.6	25	0.512320	-6.2	55
KL23	87	17.2	25	0.512293	-6.7	60
KL23	93	18.2	27	0.512289	-6.8	61
KL23	97	19.2	27	0.512306	-6.5	58

Core	Depth in core (cm)	Age (ka)	grain size mode (μm)
KL11	3	1.5	23
KL11	5	1.8	20
KL11	12	2.5	19
KL11	22	3.4	16
KL11	31	4.2	18
KL11	42	5.2	15
KL11	57	6.6	14
KL11	61	7.2	12
KL11	81	10.6	22
KL11	88	13.2	29
KL11	91	14.2	30

Supplementary Table 1| Grain sizes and the $^{143}Nd/^{144}Nd$ composition of the siliciclastic fraction in cores KL23[2] and KL11[65].

References

1. Sprenger, M. & Wernli, H. The LAGRANTO Lagrangian analysis tool – version 2.0. *Geosci. Model Dev.* **8,** 2569–2586 (2015).
2. Palchan, D., Stein, M., Almogi-Labin, A., Erel, Y. & Goldstein, S. L. Dust transport and synoptic conditions over the Sahara–Arabia deserts during the MIS6/5 and 2/1 transitions from grain-size, chemical and isotopic properties of Red Sea cores. *Earth Planet. Sci. Lett.* **382,** 125–139 (2013).
3. Nicholson, S. E. The nature of rainfall variability over Africa on time scales of decades to millenia. *Glob. Planet. Change* **26,** 137–158 (2000).
4. Tierney, J. E., deMenocal, P. B. & Zander, P. D. A climatic context for the out-of-Africa migration. *Geology* **45,** 1023–1026 (2017).
5. Palchan, D., Erel, Y. & Stein, M. Geochemical characterization of contemporary fine detritus in the Dead Sea watershed. *Chem. Geol.* **494,** 30–42 (2018).
6. Wasserburg, G. J., Jacobsen, S. B., DePaolo, D. J., McCulloch, M. T. & Wen, T. Precise determination of Sm/Nd ratios, Sm and Nd isotopic abundances in standard solutions. *Geochim. Cosmochim. Acta* **45,** 2311–2323 (1981).
7. Paillou, P. *et al.* Mapping of a major paleodrainage system in eastern Libya using orbital imaging radar: The Kufrah River. *Earth Planet. Sci. Lett.* **277,** 327–333 (2009).
8. Revel, M. *et al.* 20,000 years of Nile River dynamics and environmental changes in the Nile catchment area as inferred from Nile upper continental slope sediments. *Quat. Sci. Rev.* **130,** 200–221 (2015).
9. Armitage, S. J. *et al.* Multiple phases of North African humidity recorded in lacustrine sediments from the Fazzan Basin, Libyan Sahara. *Quat. Geochronol.* **2,** 181–186 (2007).
10. Gasse, F. *et al.* Biological remains, geochemistry and stable isotopes for the reconstruction of environmental and hydrological changes in the holocene lakes from North Sahara. *Palaeogeogr. Palaeoclimatol. Palaeoecol.* **60,** 1–46 (1987).
11. Nydal, R., Lovseth, K., Skullerud, K. E. & Holm, M. Natural Radiocarbon Measurements Iv. *Radiocarbon* **6,** 280–290 (1964).
12. Street, F. A. & Grove, A. T. Environmental and climatic implications of late Quaternary lake-level fluctuations in Africa. *Nature* **261,** 385–390 (1976).
13. Talbot, M. R. A review of the palaeohydrological interpretation of carbon and oxygen isotopic ratios in primary lacustrine carbonates. *Chem. Geol. Isot. Geosci. Sect.* **80,** 261–279 (1990).
14. WILLIAMS, M. A. J., BISHOP, P. M., DAKIN, F. M. & GILLESPIE, R. Late Quaternary lake levels in southern Afar and the adjacent Ethopian Rift. *Nature* **267,** 690–693 (1977).
15. GROVE, A. T. & GOUDIE, A. S. Late Quaternary Lake Levels in the Rift Valley of Southern Ethiopia and Elsewhere in Tropical Africa. *Nature* **234,** 403–405 (1971).
16. Berger, R. & Libby, W. F. UCLA RADIOCARBON DATES IX*. **11,** 194–209 (1969).
17. Delibrias, G., Ortlieb, L. & Petit-Maire, N. New 14C data for the Atlantic Sahara (Holocene): Tentative interpretations. *J. Hum. Evol.* **5,** 535–546 (1976).
18. Gasse, F. & Fontes, J.-C. Palaeoenvironments and palaeohydrology of a tropical closed lake (Lake

Asal, Djibouti) since 10,000 yr B.P. *Palaeogeogr. Palaeoclimatol. Palaeoecol.* **69,** 67–102 (1989).

19. Delibrias, G., Guillier, M. T. & Labeyrie, J. GIF NATURAL RADIOCARBON MEASUREMENTS VIII. **16,** 15–94 (1974).

20. Brookes, I. A. Geomorphology and Quaternary geology of the Dakhla Oasis Region, Egypt. *Quat. Sci. Rev.* **12,** 529–552 (1993).

21. Gasse, F. Evolution of Lake Abhe (Ethiopia and TFAI), from 70,000 b.p. *Nature* **265,** 42–45 (1977).

22. Gasse, E. & Street, F. A. Late Quaternary Lake-level fluctuations and environments of the northern Rift valley and Afar region (Ethiopia and Djibouti). *Palaeogeogr. Palaeoclimatol. Palaeoecol.* **24,** 279–325 (1978).

23. Pachur, H.-J. & Hoelzmann, P. Paleoclimatic implications of late quaternary lacustrine sediments in Western Nubia, Sudan. *Quat. Res.* **36,** 257–276 (1991).

24. Fontes, C. . *et al.* Freshwater to marine-like environments from Holocene lakes in northern Sahara. *Nature* **317,** 608–610 (1985).

25. Street, F. A. & Grove, A. T. Global maps of lake-level fluctuations since 30,000 yr B.P. *Quat. Res.* **12,** 83–118 (1979).

26. Erel, Y. *et al.* in *Quaternary of the Levant: Environments, Climate Change, and Humans* (eds. Enzel, Y. & Ofer, B.-Y.) (Cambridge University Press, 2017).

27. Kutzbach, J. E. & Street-Perrott, F. A. Milankovitch forcing of fluctuations in the level of tropical lakes from 18 to 0 kyr BP. *Nature* **317,** 130–134 (1985).

28. Hoelzmann, P. *et al.* Mid-Holocene land-surface conditions in northern Africa and the Arabian Peninsula: A data set for the analysis of biogeophysical feedbacks in the climate system. *Global Biogeochem. Cycles* **12,** 35–51 (1998).

29. Drake, N. A., Blench, R. M., Armitage, S. J., Bristow, C. S. & White, K. H. Ancient watercourses and biogeography of the Sahara explain the peopling of the desert. *Proc. Natl. Acad. Sci. U. S. A.* **108,** 458–62 (2011).

30. Kutzbach, J. E. & Liu, Z. Response of the African Monsoon to Orbital Forcing and Ocean Feedbacks in the Middle Holocene. *Science (80-.).* **278,** 440–443 (1997).

31. Hassan, F. A., Barich, B., Mahmoud, M. & Hemdan, M. A. Holocene Playa Deposits of Farafra Oasis, Egypt, and Their Palaeoclimatic and Geoarchaeological Significance. *Geoarchaeology An Int. J.* **16,** 29–46 (2001).

32. Gasse, F., Téhet, R., Durand, A., Gibert, E. & Fontes, J.-C. The arid–humid transition in the Sahara and the Sahel during the last deglaciation. *Nature* **346,** 141–146 (1990).

33. Mulitza, S., Schefuß, E., Lopes, R. A., Damste, J. S. S. & Schouten, S. Wet phases in the Sahara / Sahel region and human migration patterns in North Africa. **106,** 20159–20163 (2009).

34. Tjallingii, R. I. K. *et al.* Coherent high- and low-latitude control of the northwest African hydrological balance. **1,** 1–6 (2008).

35. Piotrowski, A. M., Goldstein, S. L., Hemming, S. R. & Fairbanks, R. G. Intensification and variability of ocean thermohaline circulation through the last deglaciation. **225,** 205–220 (2004).

36. Collins, J. A. *et al.* Abrupt shifts of the Sahara – Sahel boundary during Heinrich. *Clim. Past* **9,** 1181–1191 (2013).

37. Giannini, A., Biasutti, M., Held, I. M. & Sobel, A. H. A global perspective on African climate. *Clim. Change* **90,** 359–383 (2008).

38. Weldeab, S., Lea, D., Schneider, R. & Andersen, N. 155,000 years of West African monsoon and ocean thermal evolution. *Science (80-.).* **316,** 1303 (2007).

39. Gasse, F. Hydrological changes in the African tropics since the Last Glacial Maximum. *Quaternary Science Reviews* **19,** 189–211 (2000).

40. Gasse, F. & Van Campo, E. Abrupt post-glacial climate events in West Asia and North Africa monsoon domains. *Earth Planet. Sci. Lett.* **126,** 435–456 (1994).

41. Revel, M. *et al.* 100,000 Years of African monsoon variability recorded in sediments of the Nile margin. *Quat. Sci. Rev.* **29,** 1342–1362 (2010).

42. Watrin, J., Lézine, A.-M. & Hély, C. Plant migration and plant communities at the time of the "green Sahara". *Comptes Rendus Geosci.* **341,** 656–670 (2009).

43. Wendorf, F., Expedition, T. M. of the C. P. & Members of the combined prehistoric expedition. Late Pleistocene and Recent Climatic Changes in the Egyptian Sahara. *Geogr. J.* **143,** 211 (1977).

44. Haynes, C. V. J., Eyles, C. H., Pavlish, L. A., Ritchie, J. C. & Rybak, M. Holocene palaeoecology of the eastern Sahara; Selima Oasis. *Quat. Sci. Rev.* **8,** 109–136 (1989).

45. Pachur, H.-J. & Kropelin, S. Wadi Howar: Paleoclimatic Evidence from an Extinct River System in the Southeastern Sahara. *Science (80-.).* **237,** 298–300 (1987).

46. Pachur, H. & Hoelzmann, P. Late Quaternary paleoecology and paloeclimates of the eastern Sahara. *J. African Earth Sci.* **30,** 929–239 (2000).

47. Gasse, F. Diatoms for reconstructing palaeoenvironments and paleohydrology in tropical semi-arid zones. *Hydrobiologia* **154,** 127–163 (1987).

48. Quade, J. *et al.* Megalakes in the Sahara? A Review. *Quat. Res.* 1–23 (2018). doi:10.1017/qua.2018.46

49. MacDonald, A. M., Bonsor, H. C., Dochartaigh, B. É. Ó. & Taylor, R. G. Quantitative maps of groundwater resources in Africa. *Environ. Res. Lett.* **7,** 024009 (2012).

50. Tierney, J. E., Pausata, F. S. . & deMenocal, P. B. Rainfall regimes of the Green Sahara. *Sci. Adv.* **3,** (2017).

51. Wassenburg, J. A. *et al.* Reorganization of the North Atlantic Oscillation during early Holocene deglaciation. *Nat. Geosci.* **9,** 602–605 (2016).

52. Adkins, J., deMenocal, P. & Eshel, G. The 'African humid period' and the record of marine upwelling from excess 230Th in Ocean Drilling Program Hole 658C. *Paleoceanography* **21,** 1–14 (2006).

53. McGee, D., deMenocal, P. B., Winckler, G., Stuut, J. B. W. & Bradtmiller, L. I. The magnitude, timing and abruptness of changes in North African dust deposition over the last 20,000 yr. *Earth Planet. Sci. Lett.* **371–372,** 163–176 (2013).

54. Pourmand, A., Marcantonio, F. & Schulz, H. Variations in productivity and eolian fluxes in the

northeastern Arabian Sea during the past 110 ka. *Earth Planet. Sci. Lett.* **221,** 39–54 (2004).

55. Bartlein, P. J. *et al.* Pollen-based continental climate reconstructions at 6 and 21 ka: a global synthesis. *Clim. Dyn.* **37,** 775–802 (2011).

56. Jolly, D. *et al.* Biome reconstruction from pollen and plant macrofossil data for Africa and the Arabian peninsula at 0 and 6000 years. *J. Biogeogr.* **25,** 1007–1027 (1998).

57. Arz, H. W., Patzold, J. & Muller, P. J. Influence of Northern Hemisphere climate and global sea level rise on the restricted Red Sea marine environment during termination I. *Paleoceanography* **18,** 1–13 (2003).

58. Roberts, A. P., Rohling, E. J., Grant, K. M., Larrasoaña, J. C. & Liu, Q. Atmospheric dust variability from Arabia and China over the last 500,000 years. *Quat. Sci. Rev.* **30,** 3537–3541 (2011).

59. Larrasoaña, J. C., Roberts, A. P. & Rohling, E. J. Magnetic susceptibility of eastern Mediterranean marine sediments as a proxy for Saharan dust supply? *Mar. Geol.* **254,** 224–229 (2008).

60. Wassenburg, J. A. *et al.* Climate and cave control on Pleistocene/Holocene calcite-to-aragonite transitions in speleothems from Morocco: Elemental and isotopic evidence. *Geochim. Cosmochim. Acta* **92,** 23–47 (2012).

61. Jungclaus, J. H. *et al.* Climate and carbon-cycle variability over the last millennium. *Clim. Past* **6,** 723–737 (2010).

62. Rodrigo-Gámiz, M., Martínez-Ruiz, F., Chiaradia, M., Jiménez-Espejo, F. J. & Ariztegui, D. Radiogenic isotopes for deciphering terrigenous input provenance in the western Mediterranean. *Chem. Geol.* **410,** 237–250 (2015).

63. Abotalib, Z. A. *et al.* Complexity of Saharan paleoclimate reconstruction and implications for modern human migration. *Earth Planet. Sci. Lett.* **508,** 74–84 (2019).

64. El-Shenawy, M. I., Kim, S.-T., Schwarcz, H. P., Asmerom, Y. & Polyak, V. J. Speleothem evidence for the greening of the Sahara and its implications for the early human dispersal out of sub-Saharan Africa. *Quat. Sci. Rev.* **188,** 67–76 (2018).

65. Clasen, F. & Gehrke, B. Sedimentologische Analyse von Kolbenlot-Kernen aus dem Roten Meer. (Gottingen, 1990).
